# Aggressive angiomyxoma presenting with huge abdominal lump: A case report

**DOI:** 10.1186/1757-1626-1-131

**Published:** 2008-08-28

**Authors:** Sanjeev Kumar, Nikhil Agrawal, Rahul Khanna, AK Khanna

**Affiliations:** 1 Department of General Surgery, Institute of Medical Sciences, Banaras Hindu University, Varanasi – 221 005, India

## Abstract

Agressive angiomyxoma is a rare mesenchymal neoplasm. It mainly presents in females. We here present a case of angiomyxoma presenting as huge abdominal lump along with gluteal swelling. Case note is described along with brief review of literature.

## Background

Aggressive angiomyxoma is a rare mesenchymal tumor occurring predominantly in the pelvi-perineal region of females. We report such a case presented as abdominal as well as gluteal lump, a very unusual presentation. Patient underwent laparotomy and tumor was successfully excised.

## Case presentation

**Figure 1:**
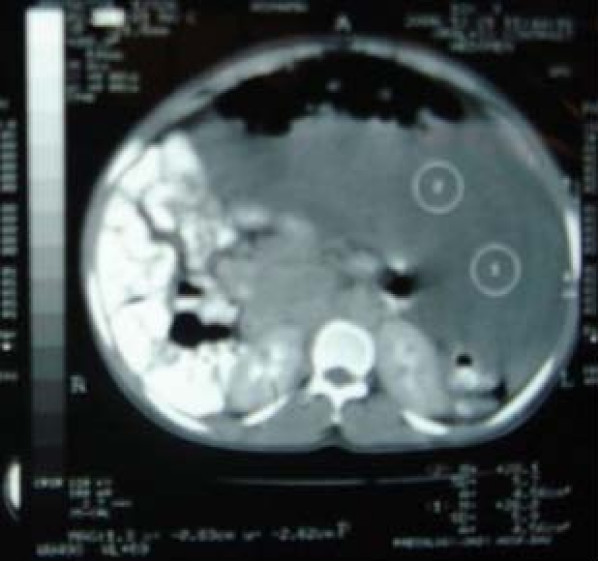
CECT of the abdomen.

An eighteen year old unmarried female presented with progressive distension of abdomen and swelling in left gluteal region for last ten months. It was associated with mild dull aching pain in lower abdomen and bilateral flanks. She was having normal menstrual history. Examination revealed distended abdomen and a non-tender, diffuse, firm and dull mass was felt all over abdomen. Free fluid in peritoneum could not be demonstrated. There was another 10 × 8 cm boggy swelling on upper postero-medial aspect of left thigh having cross fluctuation with abdominal swelling. On ultrasonography 30 × 18 × 16 cm complex cystic mass was found occupying whole abdomen and pelvis with internal septations and echoes displacing bowel loops posteriorly with mild right sided hydro-ureteronephrosis. CECT scan (Figure 1) showed heterogeneous soft tissue mass, adjacent to coccyx, involving left gluteal region and extending superiorly into pelvis and abdomen with anterolateral displacement of urinary bladder and lateral displacement of bowel with right hydronephrosis. Rectum was displaced anteriorly and to the right. The lesion was complex with variable soft tissue attenuation (+23.0 HU). Fat plane between the mass lesion and pelvic musculature and abdominal wall muscles was intact. Fine needle aspiration cytology was inconclusive and trucut needle biopsy showed round to spindle shaped cells in a loose to fibrous stroma with fair number of intervening vessels, suggestive of spindle cell tumor.

**Figure 2:**
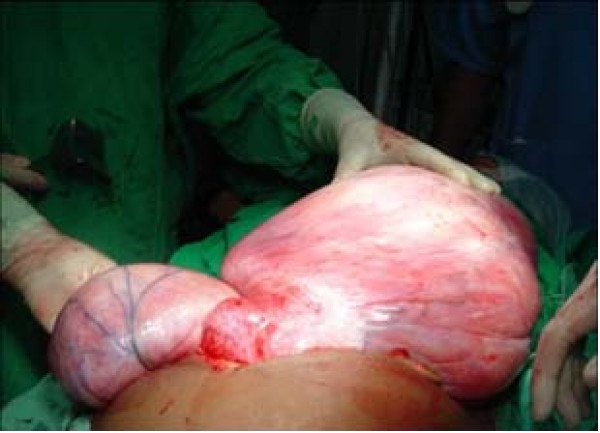
Intraoperative photograph of the tumor.

Patient underwent laparotomy and the tumor was excised. The tumor was found to be bilobed, weighing about 15 Kg. (Figure 2). Rectum was displaced anteriorly and to right. Right ureter was displaced laterally with hydroureter. Tumor was extending into thigh through left obturator foramen. Urinary bladder was adherent to mass and displaced anteriorly. Uterus and fallopian tubes were normal, tumor was found to be originating from rectovaginal septum. Liver was normal and there was no ascites or lymphadenopathy.

**Figure 3:**
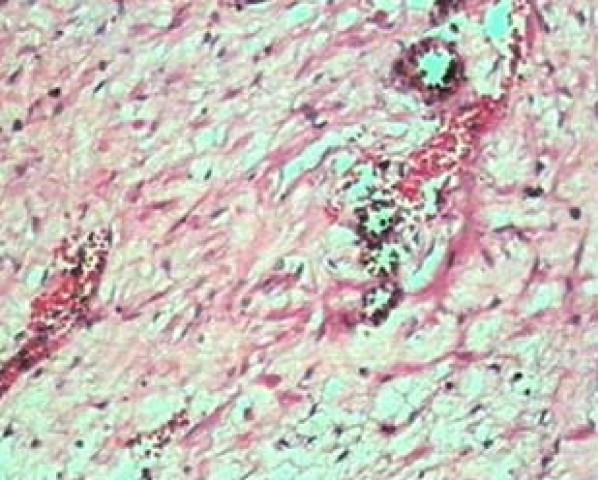
Microscopic photograph of the tumor.

The histopathology of the specimen showed capillary and cavernous vascular spaces stuffed with blood and separated by edematous fibrous and myxomatous tissue. The fibrous tissue is in form of interlacing or parallel bands of collagen with edema. The myxomatous tissue comprise of stellate cells with fibrillary in mucinous setting (Figure 3). Immuno-histochemistry was positive for vimentin and desmin while negative for actin and myosin. These findings were consistent with the diagnosis of aggressive angiomyxoma.

## Discussion

Aggressive angiomyxoma is an uncommon mesenchymal neoplasm occurring predominantly in the pelvi-perineal region of adults, first described in 1983 by Steeper and Rosai [1]. About 90% of patients are women, usually of reproductive age [2]. A few cases have been described in males, usually in scrotum. It presents as a painless, poorly circumscribed gelatinous vulvar mass and clinically simulates a bartholin gland cyst or an inguinal hernia. On gross examination the tumors are lobulated, soft to rubbery, solid masses. The cut surface reveals a glistening, soft homogeneous appearance. Recurrent tumors show more prominent areas of hemorrhage and fibrosis. Histologically angiomyxoma is a mesenchymal tumor, composed of fibroblasts within a strong myxoid background. Vascular proliferation is also prominent, and virtually no mitoses are present [3]. The vast majority of cases demonstrate positivity for desmin in the myxoid bundles and/or stromal cells, while actins and CD34 may be variably positive [3]. The estrogen and progesterone receptor positivity suggests that aggressive angiomyxoma might be hormone dependent as rapid growth has been observed during pregnancy. The tumor grows slowly, and its benign nature is suggested by the histology and by the fact that it shows no tendency to metastasize. However, it is locally aggressive and tends to recur (36–72%) after resection [4]. Imaging of these tumors is important to determine extent and, thus, the optimal surgical approach. Sonography shows a mass that is hypoechoic or appears frankly cystic. Angiography usually shows a generally hypervascular mass. It has a characteristic appearance on CT and MR imaging and these techniques reveal the extent of the tumor as well. On CT, the tumor has a well-defined margin and attenuation less than that of muscle. On T2-weighted MR imaging, the tumor has high signal intensity [4]. Treatment is usually surgery in form of wide local excision. Preoperative angiographic embolization, preoperative external beam irradiation and intraoperative electron beam radiotherapy are useful to decrease the chances of local recurrence [5]. Hormonal treatment with a gonadotropin-releasing hormone agonist can be applied for small primary aggressive angiomyxomas in addition to adjuvant therapy for residual tumors [6].

## Conclusion

Although a rare diagnosis, aggressive angiomyxoma can present with unusual features. Detailed radiological examination is helpful in suspecting the problem, but histology is gold standard for diagnosis. Wide excision is curative and prognosis of such tumor is good.

## Competing interests

The authors declare that they have no competing interests.

## Authors' contributions

AKK and RK: operating surgeons, SK: collected clinical details including photographs, summarized the case history and prepared final draft, NA: conducted a literature search and prepared first draft. All authors read and approved the final manuscript.

## Consent

A fully informed written consent was obtained from the patient for the publication of this case report and accompanying images. A copy of the written consent can be sent to Editor-in-Chief of this journal if article is accepted for publication.
